# Emergence of Treadmill Running Ability and Quantitative Assessment of Gait Dynamics in Young Ts65Dn Mice: A Mouse Model for Down Syndrome

**DOI:** 10.3390/brainsci13050743

**Published:** 2023-04-29

**Authors:** Jonah J. Scott-McKean, Ryan Jones, Mark W. Johnson, Joyce Mier, Ines A. Basten, Melissa R. Stasko, Alberto C. S. Costa

**Affiliations:** 1 Department of Macromolecular Science and Engineering, Case Western Reserve University, Cleveland, OH 44106-6090, USA; jonah.scott-mckean@case.edu; 2 College of Medicine and Life Sciences, The University of Toledo, Toledo, OH 43606-3390, USA; ryan.jones9@rockets.utoledo.edu; 3 Department of Biochemistry, Case Western Reserve University, Cleveland, OH 44106-6090, USA; 4 Department of Pediatrics, Case Western Reserve University, Cleveland, OH 44106-6090, USA; markw.johnson@roadrunner.com (M.W.J.); melissa.stasko@case.edu (M.R.S.); 5 Physical Therapy Program, University of Wisconsin, Madison, WI 53706-1532, USA; joycemier1@gmail.com; 6 Department of Biology, Case Western Reserve University, Cleveland, OH 44106-6090, USA; 7 Psychiatric Hospital Asster, 3800 Sint-Truiden, Belgium; bastenines@gmail.com; 8 Department of Psychiatry, Case Western Reserve University, Cleveland, OH 44106-6090, USA

**Keywords:** down syndrome, trisomy 21, Ts65Dn mouse, neurodevelopment, balance, motor control

## Abstract

Down syndrome (DS), which results from the complete or partial trisomy of chromosome 21 (trisomy-21), is the most common genetically defined cause of intellectual disability. Trisomy-21 also produces, or is associated with, many neurodevelopmental phenotypes and neurological comorbidities, including delays and deficits in fine and gross motor development. The Ts65Dn mouse is the most studied animal model for DS and displays the largest known subset of DS-like phenotypes. To date, however, only a small number of developmental phenotypes have been quantitatively defined in these animals. Here, we used a commercially available high-speed, video-based system to record and analyze the gait of Ts65Dn and euploid control mice. Longitudinal treadmill recordings were performed from p17 to p35. One of the main findings was the detection of genotype- and sex-dependent developmental delays in the emergence of consistent, progressive-intensity gait in Ts65Dn mice when compared to control mice. Gait dynamic analysis showed wider normalized front and hind stances in Ts65Dn mice compared to control mice, which may reflect deficits in dynamic postural balance. Ts65Dn mice also displayed statistically significant differences in the variability in several normalized gait measures, which were indicative of deficits in precise motor control in generating gait.

## 1. Introduction

Down syndrome (DS) is the phenotypic expression of the most common human chromosomal disorder compatible with live birth: a complete or partial extra copy of chromosome 21 (trisomy 21) [1]. The rate of live births with DS is dependent on maternal ages in the general population and on the extent of the reduction in live births of children with DS using elective DS-related termination of pregnancy, and as a result, will vary over time and between countries and regions. In the US, for the period 2010–2014, this rate was estimated at 1 in 732 [2]. Individuals with DS typically manifest a moderate degree of intellectual disability [3], with disproportionate deficits in the hippocampus- and prefrontal-cortex-dependent functions [4,5,6,7]. The presence of a neuropathology indistinguishable from Alzheimer’s disease (AD) in persons with DS in their mid-thirties to early forties is virtually universal [8]. Additionally, the high prevalence of early onset dementia in this population (which is almost certainly the clinical consequence of AD-like neuropathology [9]) is an issue of growing epidemiological significance [10,11].

Neurodevelopmental delays are not restricted to cognitive deficits. The development of gross and fine motor skills in persons with DS is also significantly affected when compared to neurotypical norms [12,13]. For example, the emergence of sitting, crawling, and walking skills are all significantly delayed in relation to typically developing infants and toddlers [13]. Later in development, children, adolescents, and adults with DS display longer reaction and movement times, deficits in balance and postural reflexes (such as the optokinetic and vestibular reflexes), and increased co-contraction of agonist and antagonist muscle pairs [12,13,14,15,16,17,18,19]. The neuropathologic basis for motor dysfunction in persons with DS is not well known; however, it has been suggested that cerebellar dysfunction, delayed myelination, and/or proprioceptive deficits can potentially contribute to this phenotype [20,21,22,23,24].

In most countries, habilitative services, primarily in the form of physical and occupational therapies, are provided to address motor dysfunction in children with DS. However, the level of evidence supporting the efficacy of such interventions is only weak-to-moderate due to the technical and ethical difficulties of running randomized controlled trials in the field of habilitative early intervention. For example, a recent study reports a minimal impact of advances in the management of children with DS on the age of gross motor skill acquisition. In that study, the authors state that their “findings suggest that timing of gross motor skill achievement in children with DS has remained stable despite changes in medical practice and information over the past 29 years” [13]. In this context, one can argue that the use of preclinical animal models may have a role in informing the development of specific forms of both non-pharmacological and pharmacological interventions aimed at accelerating acquisition and enhancing motor function.

Three decades after it was first described [25], the Ts65Dn mouse is still the most widely researched mouse model for DS. In spite of its shortcomings as a model for DS, due primarily to the incompleteness and partial nonspecificity in the set of human chromosome 21 orthologous genes contained in its trisomic segment [26], the Ts65Dn mouse has proven helpful in recapitulating many features of DS and as a tool to predict phenotypes previously not known in persons with DS. These DS phenotypes first found in mice included hippocampus-dependent cognitive deficits [27] and specific neuroanatomic alterations in the cerebellum [7]. The Ts65Dn mouse also displays several other DS-relevant phenotypic features [27]. For instance, previous studies have shown that adult Ts65Dn mice have motor dysfunction and abnormalities in gait when compared to euploid mice [12,28]. At present, however, only a small number of developmental phenotypes (all rodent-specific) have been quantitatively defined in these animals [29,30].

In the present study, we used a commercially available high-speed, video-based, computerized, treadmill system (DigiGait^®^, Mouse Specifics, Inc., Quincy, MA, USA) to longitudinally study the emergence of gait and the gait dynamics in Ts65Dn mice. Our two main goals were (1) to test whether, similar to individuals with DS, Ts65Dn mice exhibit delayed development of motor skills compared to euploid control mice and (2) to quantify gait parameters in Ts65Dn mice that underwent mild-to-moderate degrees of physical training on the treadmill system.

## 2. Materials and Methods

### 2.1. Mice

The original production of the segmental trisomy Ts65Dn has been well described in the literature [25]. Experimental mice were generated with repeated backcrossing of Ts65Dn females to C57BL/6JEiJ × C3Sn.BLiA–Pde6b+/D F1 hybrid males (as described in [31]) at the Case Western Reserve University’s Animal Resource Center. A total of 59 mice were used in this study (Ts65Dn male (*n* = 13) and female mice (*n* = 13); and euploid male (*n* = 21) and female mice (*n* = 12)). Animals were kept in a 12 h light and dark schedule with ad libitum access to food and water. All experimental procedures and protocols were in strict compliance with the Guide for the Care and Use of Laboratory Animals Eighth Edition (National Institutes of Health, The National Academies Press, Washington, DC, USA) and were approved by the Institutional Animal Care and Use Committee (IACUC) at CWRU.

### 2.2. Gait Dynamics Recording

A DigiGait^®^ analysis system (Mouse Specifics, Cambridge, MA, USA) was utilized to record the gait of Ts65Dn and euploid littermate control mice. The compartment of the treadmill in which the mouse runs is ∼25 cm in length and ∼5 cm wide and can be changed to accommodate different-sized animals [12]. Digital video images showing the ventral view of mice were obtained with a high-speed camera that recorded running on a motorized transparent treadmill belt. The camera collected images at 181 frames per second (i.e., 5.52 ms time resolution) that were stored in Audio Video Interleaved (AVI) format onto a hard drive. In this study, approximately ten strides of imaging data were stored for each mouse while running at both 18 cm/s and 36 cm/s during each testing day and age. Offline, DigiGait^®^ generates a colored graphical representation of the paw placement within each image frame, which is analyzed to generate the axial (x) and lateral (y) position of each paw in a moving reference frame during walking or running.

### 2.3. Treadmill Longitudinal Training Procedure

Starting at p17, Ts65Dn male (*n* = 13) and female (*n* = 12) mice, along with euploid male (*n* = 21) and female (*n* = 13) mice, were run on the treadmill. (Note that the imbalance in the number of euploid and Ts65Dn male mice was due to the fact that animals were PCR-genotyped only after the experiments were concluded, and some of the euploid male mice were simply misclassified using inspection during group selection before the experiments.) Each mouse was tested on every second day (at ages p17, p19, … p35). All mice were weighed before being placed within the DigiGait^®^ apparatus. On each day, the mouse was allowed to acclimate to the treadmill with the belt not moving (0 cm/s) for 5 min, then it was presented with treadmill speeds of 8, 12, 16, and 18 cm/s for 5 s with a 5-second break in between rates. The camera was not recording during this acclimatization period. If, at any point, the mouse was not able to complete a specified speed, the acclimation period was ended. The speed was set at 18 cm/s or the maximum speed the mouse could achieve, and a video was recorded with the mouse running for up to 30 s. Approximately ten strides of video data were saved where the mouse did not stumble or pause on the treadmill. After completing the first round, each mouse that had not reached its maximum speed was exposed to higher speeds of 22, 26, 30, 34, and 36 cm/s for 5 seconds with 5-second breaks in between. Finally, a video was recorded at the maximum speed attained by each mouse for up to 30 s, saving approximately ten strides of video data. Consistent with previous work [12], speeds above 36 cm/s were not utilized in this study. (See Supplementary Videos S1–S4 for representative examples of mice running on the treadmill.)

### 2.4. Paw Classification Analysis

**Figure 1:**
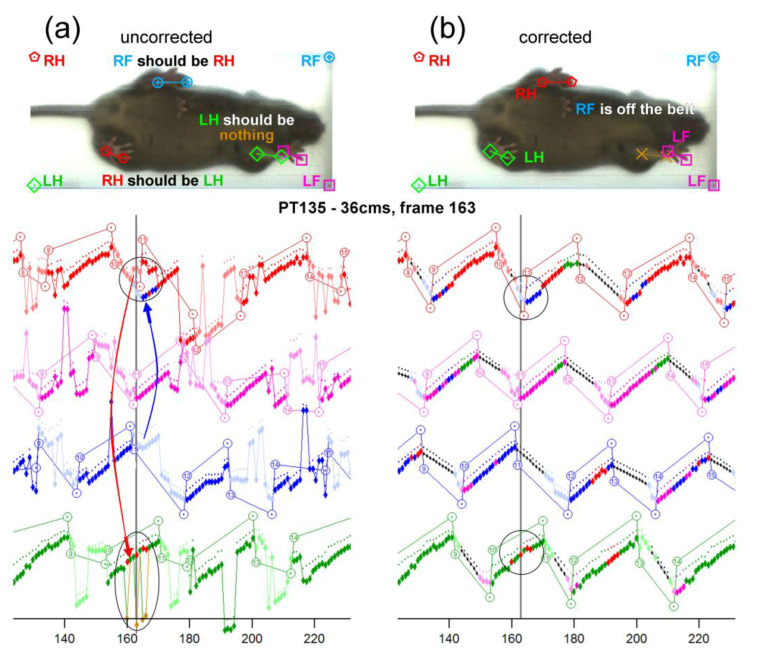
Custom-programmed software from IgorPro that allows for each frame to be corrected. Initial analysis using DigiGait^®^ with many improperly classified paw placements. (**a**) As seen in the frame, the left hind paw is categorized as the right hind paw. Furthermore, the right hind paw is categorized as the right front paw. Red is the right hind paw, blue is the right front paw, pink is the left front paw, and green is the left hind paw. (**b**) A frame-by-frame correction was necessary to repair the step traces. Looking at the graph underneath, the circles highlight some of the modifications that had to be made to fix this recording. The repaired-step charts were utilized to find the different required measurements.

Each video was processed using the DigiGait^®^ analysis software, as described previously [12]. However, further analysis was needed upon completion of the DigiGait^®^ software analysis to properly classify mouse paws. Sometimes the DigiGait^®^ analysis software would improperly classify paws, including detecting the nose, tail, genitals, and the wrong paws (Figure 1a). This led to our manually correcting each of the videos with custom-programmed software made in IgorPro (WaveMetrics, Inc., Portland, OR, USA). Editing corrections were made semi-manually as needed upon reviewing graphical traces, cursors, and mouse images with overlaid paw detection graphics (Figure 1b). Using this procedure, approximately 7.0% of all the paw placements had to be edited. The following gait parameters were obtained: stride length, lateral shift, hind and front stance width, front/hind skew, front/hind distance, hind/front offset, stride interval, and swing percentage.

### 2.5. Statistical Analyses

Results are presented as mean ± SEM. Data on motor development assessed using the emergence of the ability to consistently run on the treadmill were analyzed with non-linear fitting sigmoidal curves (extra sum of squares F tests for the day the animal cohort reached 50% success) and reverse-survival curves (log-rank, Mantel–Cox test) in GraphPad Prism (version 7.0, GraphPad Software, La Jolla, CA, USA). Comparisons between the means for gait dynamic measures from Ts65Dn vs. euploid control mice were performed using factorial analysis of variance (ANOVA) in Statistica Academic (Statistica, version 13, TIBCO Software, Palo Alto, CA, USA). Treadmill speed, genotype, and sex were the categorical factors. Given that the primary goal of this study was to investigate genotype-dependence, when a significant genotype-dependence was detected (even in the absence of a detectable treadmill-speed- or sex-dependence), a complete set of Fisher’s LSD pairwise post hoc comparisons was performed to determine significance. A *p*-value of <0.05 was considered statistically significant. In all figures, *p*-values of <0.05, 0.01, and 0.001 were represented with *, **, and ***, respectively.

## 3. Results

### 3.1. Delay in Motor Development

**Figure 2:**
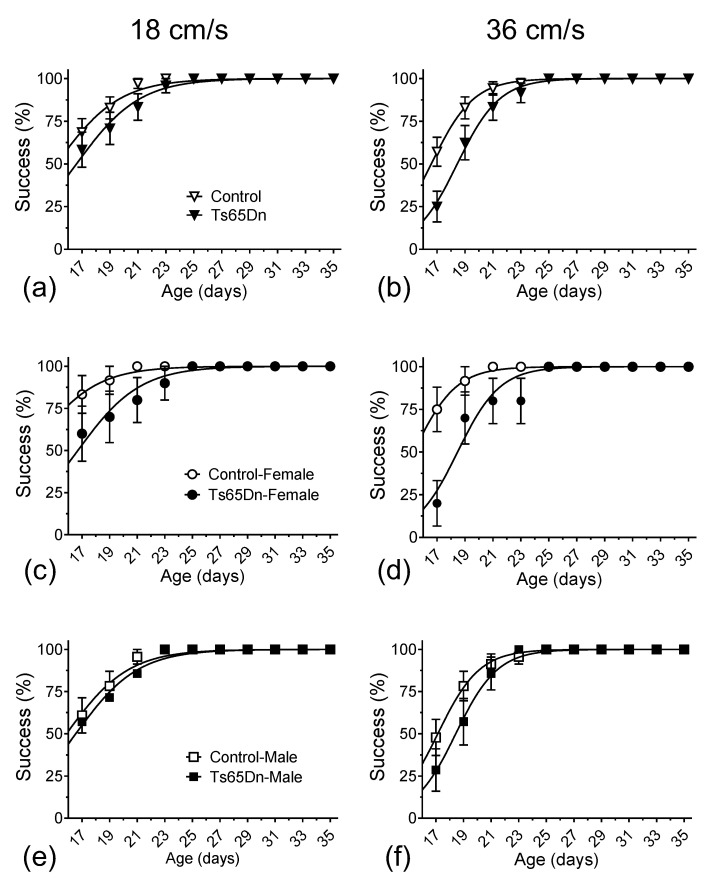
The percentage of mice able to reach designated speeds vs. age. Data were non-linearly fitted with sigmoidal curves. Both Ts65Dn and control mice eventually reached the target speeds of 18 and 36 cm/s. (**a**) Ts65Dn mice were delayed reaching 18 cm/s by 1.5 days compared to euploid control mice. (**b**) Ts65Dn mice were delayed reaching 36 cm/s by 1.9 days compared to euploid control mice. When specifically comparing female Ts65Dn mice to female control mice, the delays in the emergence of consistent treadmill running were 4.3 days at 18 cm/s (**c**) and 3.2 days at 36 cm/s (**d**). In contrast, we observed only a non-statistically significant 0.6-day delay in male Ts65Dn mice when compared to male control mice at 18 cm/s (**e**) and a small, but statistically significant delay of 1.3 days at 36 cm/s (**f**). Data are expressed as mean ± SEM.

During the course of the treadmill training/recording sessions, which took place every other day from p17 to p35, both Ts65Dn and euploid mice were eventually able to run consistently at 18 and 36 cm/s. Still, we observed a delay in the mean age at which Ts65Dn mice reached the ability to run at these target speeds when compared to euploid littermate control mice. The mean delay for all Ts65Dn mice (i.e., when we combined data from female and male mice) vs. all control euploid mice was 1.5 days when running at 18 cm/s (Figure 2a) and 1.9 days at 36 cm/s (Figure 2b). We used two different analytical approaches to quantify the statistical significance of this delay. First, and primarily, data were analyzed using non-linear fitting with a sigmoidal curve (solid lines in Figure 2a,b), with an extra sum of squares F tests for the day the animal cohort reached 50% success, which resulted in the following *p*-values: 0.0013 and <0.0001, for the 18 cm/s and 36 cm/s datasets, respectively. Second, we used a simple reverse-survival curve (log-rank, Mantel–Cox test), which resulted in *p* = 0.3164 and *p* = 0.0105, for the 18 cm/s and 36 cm/s datasets, respectively. Therefore, both approaches detected statistically significant delays in motor development in Ts65Dn mice compared to euploid control mice.

**Figure A1:**
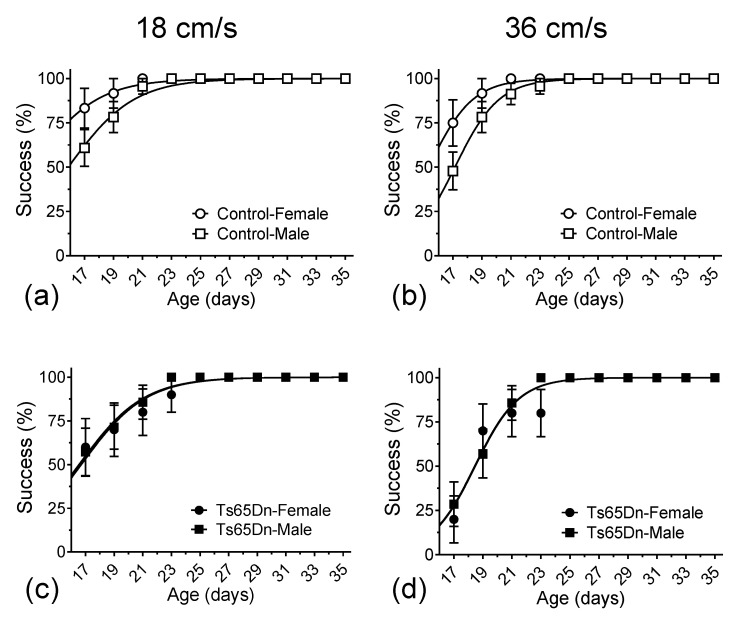
The percentage of mice able to reach designated speeds vs. age. Data were non-linearly fitted with sigmoidal curves. Euploid control male mice were delayed reaching 18 cm/s by 2.1 days compared to euploid control female mice when the treadmill was set at 18 cm/s (**a**) and by 1.9 days at 36 cm/s (**b**) In contrast, we observed no statistically significant delay between female and male Ts65Dn mice at neither 18 cm/s (**c**) nor 36 cm/s (**d**). Data are expressed as the mean ± SEM.

When the data were further parsed by sex (Figure 2c–f), we noticed that most of the genotype differences seen in Figure 2a,b could be interpreted as the result of the observation that female control mice were precocious in their ability to run consistently at 18 cm/s and 36 cm/s speeds. Indeed, after applying the same non-linear fitting analysis to compare the postnatal ages of 50% success of euploid control female and euploid control male mice to run on the treadmill at 18 cm/s and 36 cm/s, statistically significant differences were observed between the mean values recorded from these two groups (14.15 and 16.29 days at 18 cm/s, *p* = 0.0001, and 15.26 and 17.11 days at 36 cm/s, *p* < 0.0001, respectively) (Figure A1a,b in Appendix A). In contrast, no significant sex-dependence for the postnatal age of 50% consistent treadmill-running success was observed between female and male Ts65Dn mice (16.47 and 16.27 days at 18 cm/s, *p* = 0.7961, and 18.46 and 18.44 days at 36 cm/s, *p* = 0.9582, respectively) (Figure A1c,d in Appendix A).

### 3.2. Gait Dynamics

**Figure 3:**
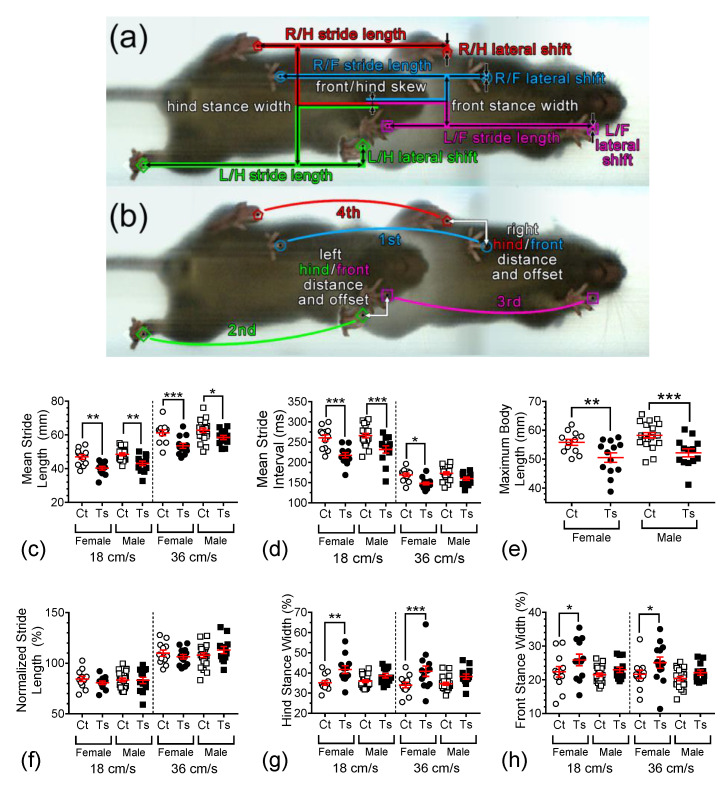
(**a**,**b**) Two overlaid video images that are 32 frames or 176.8ms apart that exhibit all four paws being down to illustrate the gait parameters analyzed here. Scatter-plot graphs in the lower section summarize important parameters by group: (**c**) stride length, (**d**) stride interval, (**e**) body length, (**f**) normalized stride length, and (**g**) hind and (**h**) front stance widths. In these graphs, individual data points are shown with overlaid red bars representing mean ± SEM. Brackets and asterisks above the data values indicate statistical significance, with *, *p* < 0.05; **, *p* < 0.01; and ***, *p* < 0.001.

Figure 3a,b show two overlaid video images that are 32 frames (or 176.8 ms) apart, both of which exhibit all four paws being down to show the different gait parameters analyzed here. Measurements of stride length, lateral shift, hind and front stance width, and front/hind skew were obtained (Figure 3a). Stride length is the distance that each foot moves on the treadmill between successive steps. Lateral shift is how far in the sideways direction each paw moves between successive steps. Front and hind stance widths are the distances between the front paws and the hind paws, respectively. Front/hind skew is a measure of lateral misalignment between the front and hind paws, which will increase in variability when the mouse shifts left and right while running. The left and right hind/front distances and offsets measure how close the hind paws are placed from the front paw locations, along with the swing of each paw (Figure 3b). The stride interval is the time from each paw placement to the subsequent placement of the same paw. The hind/front distance and offset are the axial and lateral distance between the front and hind ipsilateral paw placements.

Figure 3c depicts mean raw stride lengths measured at 18 and 36 cm/s for the four groups of animals investigated in this study. Raw stride length was found to be significantly dependent on treadmill speed (F_1,108_ = 228.26, *p* < 0.0001), genotype (F_1,108_ = 40.50, *p* < 0.0001), and sex (F_1,108_ = 8.10, *p* = 0.0053). Post hoc tests revealed significant differences between the mean raw stride length of female Ts65Dn when compared to female control mice at both 18 and 36 cm/s (*p* = 0.0013 and *p* = 0.0002, respectively). Similarly, significant differences in this measure were observed between male Ts65Dn and male control mice at both 18 and 36 cm/s (*p* = 0.0025 and *p* = 0.0200, respectively). Post hoc comparisons between mice with the same genotype but different sex produced somewhat inconsistent results. Between female and male Ts65Dn mice running at 18 cm/s, no significant differences were detected (*p* = 0.1596), whereas a significant difference was detected between these two groups when the treadmill speed was set at 36 cm/s (*p* = 0.01386). Between female and male euploid control mice running at both 18 and 36 cm/s, no significant differences were detected (*p* = 0.3945 and *p* = 0.4187, respectively).

The mean stride interval is shown in Figure 3d for the four groups of mice. This gait parameter is inversely related to mean raw stride interval, and, accordingly, was also found to be significantly dependent on treadmill speed (F_1,108_ = 383.973, *p* < 0.0001), genotype (F_1,108_ = 44.571, *p* < 0.0001), and sex (F_1,108_ = 4.433, *p* = 0.0376). Additionally, a significant interaction between speed and genotype was detected (F_1,108_ = 6.551, *p* = 0.0119). Post hoc tests revealed significant differences between the mean raw stride interval of female Ts65Dn when compared to female control mice at 18 and 36 cm/s (*p* < 0.0001 and *p* = 0.0167, respectively). Significant differences were also found between the mean raw stride length of male Ts65Dn when compared to male control mice at 18 cm/s (*p* < 0.0001), but not at 36 cm/s (*p* = 0.1013). No significant differences were detected between female and male Ts65Dn mice running at both 18 and 36 cm/s (*p* = 0.1073 and *p* = 0.1828, respectively), which was also the case for female and male euploid control mice running at these speeds (*p* = 0.4774 and *p* = 0.6555, respectively).

Changes in mouse body length can significantly affect gait metrics [32]. In previous work, it was reported that the mean weight and body length of Ts65Dn mice are significantly smaller than euploid mice [31]. Here, we confirmed that genotype (F_1,55_= 20.020, *p* <0.0001), but not sex (F_1,55_ = 2.660, *p* = 0.1086), has a statistically significant effect on nose-to-base length (Figure 3e). Both female and male Ts65Dn mice were found to be significantly shorter in length than their control counterparts (*p* = 0.0076 and *p* = 0.0006, respectively). Accordingly, we used this information to interpret the genotype dependence of various gait parameters.

When we normalized the stride length data by dividing the individual mean of the stride length for each animal by the maximum body length, using calibrated video images in the same running session, we observed that neither genotype (F_1,108_ = 0.14, *p* = 0.7101) nor sex (F_1,108_ = 0.65, *p* = 0.4217) had a statistically significant effect on this normalized measure (Figure 3f). As one would reasonably expect, however, the mean stride length remained dependent on treadmill speed even when normalized (F_1,108_ = 202.05, *p* < 0.0001).

**Figure A2:**
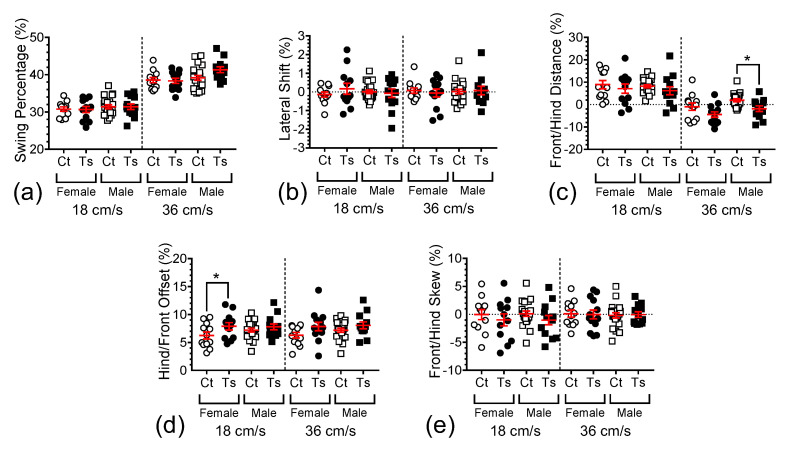
Graphical depictions of several normalized gait dynamics measures. The significance of a normalized parameter was obtained using a two-way ANOVA test. Significances between groups were then acquired using a Fisher’s LSD post hoc analysis. The brackets above indicate significance in the manner of * *p* < 0.05. The graphs show (**a**) swing percentage, (**b**) lateral shift, (**c**) front/hind distance, (**d**) hind/front offset, and (**e**) front/hind skew. Data are expressed as the mean ± SEM. *, *p* < 0.05.

Using this procedure to normalize five additional spatial gait dynamics measures (lateral shift, front/hind distance, hind/front offset, and front/hind skew) produced similar results (Figure A2 and Table A1 in Appendix A), i.e., complete or near-complete abolition of genotype-dependence in these measures when body length is taken into consideration. However, two gait parameters that may be related to general balance, the hind and front stance widths, remained robustly genotype-dependent after normalization.

Hind stance width (Figure 3g) was found to be significantly dependent on genotype (F_1,108_ = 26.480, *p* < 0.0001), but not on treadmill speed (F_1,108_ = 0.878, *p* = 0.3509) or sex (F_1,108_ = 1.200, *p* = 0.2757). Post hoc analysis showed that mean hind stance was significantly wider in Ts65Dn female mice vs. control euploid female mice at 18 and 36 cm/s (*p* = 0.0018, *p* = 0.0008, respectively), but no significant difference was detected between Ts65Dn male mice and control euploid male mice at either treadmill speed (*p* = 0.1328, *p* = 0.0516, respectively). In contrast, front stance width (Figure 3h) was found to be both significantly dependent on genotype (F_1,108_ = 10.572, *p* = 0.0015) and sex (F_1,108_ = 6.574, *p* = 0.0117) but not on treadmill speed (F_1,108_ = 1.252, *p* = 0.2656). Again, post hoc comparisons showed the mean front stance to be significantly wider in Ts65Dn female mice vs. control euploid female mice at 18 and 36 cm/s (*p* = 0.0384, *p* = 0.0366, respectively), whereas no significant difference was detected between Ts65Dn male mice and control euploid male mice at either treadmill speed (*p* = 0.3298, *p* = 0.2341, respectively). For female vs. male Ts65Dn mice running at 18 and 36 cm/s, no significant difference was detected (*p* = 0.1340 and *p* = 0.1879, respectively). Similarly, for female vs. male euploid control mice running at 18 and 36 cm/s, no significant difference was detected (*p* = 0.6523 and *p* = 0.7091, respectively).

### 3.3. Variability in Gait Dynamics Measures

To study the variability in gait dynamics measures, the standard deviations of nine normalized parameters were obtained. The variability measures show the degree to which each parameter changes within each trial and therefore represent measures of the precision of motor control.

**Figure 4:**
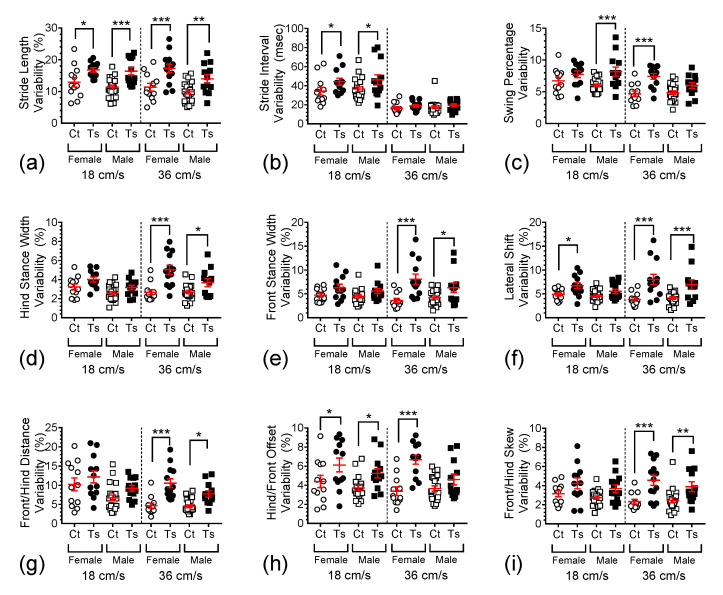
Scatter-plot depiction showing the differences in variability for all the parameters. The graphs represent variability in (**a**) stride length, (**b**) stride interval, (**c**) swing percentage, (**d**) hind stance width, (**e**) front stance width, (**f**) lateral shift, (**g**) front/hind distance, (**h**) hind/front offset, and (**i**) front/hind skew. In these graphs, individual data points are shown with overlaid red bars representing mean ± SEM. Brackets and asterisks above the data values indicate statistical significance, with *, *p* < 0.05; **, *p* < 0.01; and ***, *p* < 0.001.

For example, stride length variability (Figure 4a) was found to be significantly dependent on genotype (F_1,108_ = 39.862, *p* <0.0001) and sex (F_1,108_ = 5.041, *p* = 0.0268) but not on treadmill speed (F_1,108_ = 2.643, *p* = 0.1069). Post hoc comparisons revealed significant differences between the mean raw stride length of female Ts65Dn when compared to female control mice at both 18 and 36 cm/s (*p* = 0.0182 and *p* = 0.0002, respectively), which also remained true for male Ts65Dn vs. male control mice at both 18 and 36 cm/s (*p* = 0.0004 and *p* = 0.0050, respectively). Additionally, no significant difference was detected between female and male Ts65Dn mice running at 18 cm/s (*p* = 0.8036), but a significant difference between these two groups emerged when the animals ran at 36 cm/s (*p* = 0.0373). For female vs. male euploid control mice running at 18 and 36 cm/s, no significant difference was detected (*p* = 0.2644 and *p* = 0.3172, respectively).

The analysis of stride interval variability (Figure 4b) revealed significant dependence on treadmill speed (F_1,108_ = 116.3828, *p* < 0.0001) and genotype (F_1,108_ = 8.4848, *p* = 0.0044), but not sex (F_1,108_ = 0.5630, *p* = 0.4547). Post hoc comparisons of female Ts65Dn vs. female control mice, and between male Ts65Dn vs. male control mice, at 18 cm/s, detected significant differences in stride interval variability (*p* = 0.0256 and *p* = 0.0129, respectively). At 36 cm/s, no significant difference between the means of this measure was found true for female Ts65Dn and female control mice or male Ts65Dn and male control mice (*p* = 0.5554 and *p* = 0.6395, respectively). Additionally, no significant differences were detected between female and male Ts65Dn mice running at 18 and 36 cm/s, (*p* = 0.6552 and *p* = 0.8862, respectively). Similarly, for female vs. male euploid control mice running at 18 and 36 cm/s, no significant differences were detected (*p* = 0.5598 and *p* = 0.7320, respectively).

In addition to the results described in narrative form for panels (a) and (b) in Figure 4, we found significant genotype dependence for all measures of variability assessed. Table 1 summarizes the ANOVA statistics and post hoc comparison results for the measures depicted in panels (c)–(i) in this figure.

## 4. Discussion

In the present study, we used a commercially available high-speed, video-based treadmill system to record and analyze the development of gait and gait dynamics in Ts65Dn mice as compared to euploid control mice. Our main findings for Ts65Dn mice vs. control mice were (1) a significant delay in the emergence of the ability to generate consistent gait on the treadmill when compared to euploid control mice, which mirrors the delayed gross motor skill development observed in infants and toddlers with DS; (2) alterations in gait dynamics that may indicate deficits in dynamic balance; and (3) increased variability in gait parameters, which were consistent with poorer motor control. The longitudinal approach we used to quantify the developmental phenotype is likely to produce training effects similar to those observed in most behavioral tests in which a variable is measured repeatedly. On the other hand, this approach resulted in conditions closer to those experienced by persons with and without DS than it would be possible in a cross-sectional study involving a single or very few recording sessions with mice fresh out of their housing boxes.

In a study published over a decade ago, we used an earlier version of the DigiGait^®^ system, in collaboration with Drs. Thomas Hampton, Ajit Kale, and Ivo Amende, to analyze gait dynamics parameters recorded from 10 to 12-week-old Ts65Dn male mice [12]. In that cross-sectional study, we found that most observed differences in stride dynamics parameters between Ts65Dn and euploid control mice were statistically significant at a treadmill speed of 36 cm/s but not 18 cm/s. We also found that, at 36 cm/s, stride length was shorter in Ts65Dn mice and stride frequency was higher in Ts65Dn compared to control mice. In addition, hind limb swing duration was prolonged in Ts65Dn mice compared to control mice.

Here, we have taken advantage of the increased spatial and temporal resolution afforded with an updated DigiGait^®^ system to obtain an improved quantification of these parameters in male and female Ts65Dn mice that were backcrossed onto a genetic background free of the retinal degeneration mutation Pde6b^rd1^ [31]. Furthermore, we used a longitudinal study design in which the animals had ample opportunity to acclimate to the treadmill environment and, in this process, were allowed to experience mild-to-moderate physical training.

Although the DigiGait^®^ analysis software has improved greatly compared to its original version, we still noticed a significant number of misclassified paws in the analysis. This led us to develop custom-programmed software to allow us to semi-manually correct each of the videos, which resulted in editing approximately 7.0% of all the paw placements. Such a percentage of misclassified paws when using the DigiGait^®^ analysis software might not significantly affect the results of large-scale screening assessments of mutant mice with moderate-to-severe motor phenotypes. However, the limited precision of this software is likely to affect the quantification of mild or subtle phenotypes. The use of our custom-programmed, paw-classification software was still a tedious and time-consuming process, which made it prohibitive for us to generate a broader developmental chart for all gait parameters during the treadmill training sessions. Hopefully, new developments in the field of artificial intelligence will greatly increase the precision and throughput in the analysis of gait dynamic data obtained from the DigiGait^®^ treadmill system and similar commercially available data acquisition hardware.

As aforementioned, the longitudinal approach used here allowed us to identify a new and promising developmental phenotype in Ts65Dn mice. This comprised 1.5- and 1.9-day delays in the emergence of consistent gait in Ts65Dn mice when compared to euploid control mice (at 18 and 36 cm/s treadmill speeds, respectively). The analysis of the resulting data, however, also showed one of the limitations in the way we designed these experiments, which was the fact that we chose p17 as the age to start recording the emergence of gait on the treadmill. This age was initially chosen because it was the time point at which approximately 50% of Ts65Dn mice were able to consistently run at the low setting speed of 18 cm/s. (Coincidently, this was also the age at which approximately 50% of the control mice were able to run at the speed setting of 36 cm/s.) In retrospect, we should have chosen a younger age, such as p14 (i.e., an age at which most mice were not able to run consistently even at 18 cm/s), to produce more low-percentage data points (including initial running time points). This would have enhanced the fitting of reverse survival curves and non-linear sigmoidal fitting, which would have added a higher degree of precision to the quantification of the genotype dependence in the capability to reach the two set speeds, and therefore represent a limitation in this study. Future research with the Ts65Dn mouse, other mouse models for DS, and mouse models for other neurodevelopmental/neurological disorders should take advantage of this lesson.

As for gait dynamic parameters, we confirmed the findings of Hampton et al. [12] by showing that the mean stride length of Ts65Dn mice was significantly shorter than that of euploid control mice. However, when we normalized stride length by body length, to take into account the fact that the bodies of control mice are significantly longer than those of Ts65Dn mice, the significance of the genotype-dependence in this measure disappeared. We also confirmed the finding of a significantly shorter stride interval (which is the inverse of stride frequency) in Ts65Dn mice as compared to euploid control mice. The genotype dependence in this measure, nevertheless, is also likely related to the shorter bodies of Ts65Dn mice.

The genotype dependence in two other measures (front and hind stance widths) persisted after normalization, with Ts65Dn mice displaying significantly greater values when compared to control mice. One can reasonably speculate that these two measures of wider stance observed in Ts65Dn vs. control euploid mice are likely related to deficits in dynamic balance in these chromosomally altered mice.

Ts65Dn mice also displayed statistically significant increases in the mean value of the variability in gait dynamics uniformly across several measures (i.e., stride length, lateral shift, hind and front stance width, front/hind skew, front/hind distance, hind/front offset, and swing percentage). These observations are consistent with poorer motor control during gait in Ts65Dn mice in comparison to their euploid counterparts.

By far, the most striking example of sex dependence in a functional measure seen here was the observation of precocious emergence of the ability to consistently run on the treadmill by female control euploid mice. However, significant sex dependences were also detected in the ANOVA for stride length and stride interval as well as for the variability in stride length, hind stance width, front/hind distance, and hind/front offset. For Ts65Dn mice, post hoc pairwise comparisons detected significant female vs. male differences between the means of seven measures (mean raw stride length, mean stride interval, front stance width, variability in swing percentage, variability in hind stance width, variability in front/hind distance, and variability in hind/front offset). In contrast, a significant sex difference was only detected between the means of one gait dynamics measure (variability in front/hind distance) for euploid control mice. These findings reinforce the evolving consensus on the importance of using animals of both sexes when performing experiments with mouse models for DS [33,34,35,36,37]. However, the full appreciation for the potential translational importance of these observations may have to wait for similar comparative data analyses to be generated by human gait dynamic labs. One example of detectable sex-dependence in gross motor skills in children with DS comes from one of the recent studies mentioned here [13], which found that (without correcting for multiple comparisons) female children with DS achieved several gross motor skill milestones earlier than male children with DS.

As mentioned in the introduction, in spite of its shortcomings, the Ts65Dn mouse is still the most widely researched mouse model for DS and the one with the largest subset of verified DS-like phenotypes. Recently, however, newer models with greater construct validity than the Ts65Dn mice and potentially similar subsets of DS-like phenotypes have been created. One of such models is the Ts66Yah mouse, in which the region on mouse chromosome 17 not orthologous to any region on human chromosome 21, which exists in the Ts65Dn marker chromosome, was removed using chromosomal engineering [38]. Another example of a promising new mouse model for DS is the Tc(HSA21q;MAC)1Yakaz (“TcMAC21”) mouse, in which the long arm of human chromosome 21 was “cloned” as a mouse artificial chromosome (MAC). The MAC was introduced stably into embryonic stem cells which were then used to produce this mouse model [39]. The TcMAC21 mouse is not mosaic and contains 93% of the Chr. 21 protein-coding genes. At present, none of these new mouse models are widely available to the scientific community. However, when they do become available, it will be important to reproduce the research findings reported here with these animals.

The methods and data reported here represent a significant addition to the available tools that foster a better understanding of DS neurobiology. The delay in the acquisition of the ability to run consistently on a treadmill is the first developmental phenotype that can be directly related to a human phenotype, i.e., the delay in the emergence of crawling and walking skills in babies and toddlers with DS vis-à-vis typically developing infants and toddlers. In addition, the larger values of front and hind stance widths in Ts65Dn mice are potentially associated with deficits in dynamic balance, and the uniform increases in the variability in gait dynamics across several measures may indicate poorer motor control during gait. All these measures can also be seen as potential endpoints for future preclinical studies on pharmacological and non-pharmacological interventions aimed at enhancing the developmental trajectory and motor function of individuals with DS.

## 5. Conclusions

The present study, which examined the emergence of gait and gait dynamics in Ts65Dn mice when compared to control euploid mice, demonstrates that Ts65Dn mice present a significant delay in acquiring the ability to walk and run consistently on a treadmill. In addition, we observed alterations in gait dynamics in Ts65Dn mice suggesting deficits in dynamic balance and increased variability in gait parameters, which were consistent with poorer motor control in these chromosomally altered mice when compared to euploid control mice. Significant sex dependence in several measures was observed, which reinforces the importance of using animals of both sexes when performing similar experiments. These findings fill an important gap in terms of describing new quantitative Down syndrome-like phenotypes in a mouse model for Down syndrome. They represent a significant addition to the available tools that foster a better understanding of Down syndrome neurobiology and are also suitable to be used as endpoints in preclinical studies on pharmacological and non-pharmacological interventions aimed at enhancing the developmental trajectory and motor function of individuals with Down syndrome.

## Figures and Tables

**Table 1:** Factorial ANOVA statistics and post hoc comparison results for the measures depicted in panels (c)–(i) in Figure 4. Significant genotype dependence was found for all these measures of variability. All *p*-values < 0.05 are written in bold.

Measure Variability Figure 4 (Panel)	ANOVA Results for Each Dependent Variable *F*_1,108_; *p* =			Post Hoc Pairwise Comparisons *p* = @18cm/s; *p* = @36cm/s			
	Speed	Genotype	Sex	Ts65Dn Female vs. Control Female	Ts65Dn Male vs Control Male	Ts65Dn Female vs. Ts65Dn Male	Control Female vs. Control Male
Swing Percentage (c)	20.193; **<0.0001**	31.408; **<0.0001**	1.412; 0.2373	0.1334; **<0.0001**	**0.0003**; 0.0577	0.5106; **0.0349**	0.2429; 0.7638
Hind Stance Width (d)	1.777; 0.1853	32.840; **<0.0001**	11.826; **0.0008**	0.0760; **<0.0001**	0.1138; **0.0148**	0.0533; **0.0022**	0.0931; 0.9370
Front Stance Width (e)	0.3965; 0.5302	28.1412; **<0.0001**	1.5217; 0.2200	0.0918; **<0.0001**	0.1705; **0.0141**	0.4371; 0.0502	0.7727; 0.4601;
Lateral Shift (f)	0.6177; 0.4336	36.9112; **<0.0001**	2.1496; 0.1455	**0.0391**; **<0.0001**	0.1753; **0.0004**	0.1739; 0.2009	0.6265; 0.7391
Front/Hind Distance (g)	15.275; **0.0002**	23.6809; **<0.0001**	11.559; **0.0009**	0.1896; **0.0001**	0.0547; **0.0167**	**0.0428**; 0.0575	**0.0087**; 0.9154
Hind/Front Offset (h)	0.8000; 0.3731	31.7336; **<0.0001**	6.3541; **0.0132**	**0.0172**; **<0.0001**	**0.0145**; 0.1366	0.2161; **0.0035**	0.2871; 0.6681
Front/Hind Skew (i)	0.2981; 0.5862	28.7693; **<0.0001**	1.9632; 0.1640	0.0573; **0.0001**	0.0543; **0.0061**	0.3222; 0.2297	0.4087; 0.7563

## Data Availability

All other data will be made available upon reasonable request.

## Supplementary Materials

The following supporting information can be downloaded at: https://www.mdpi.com/article/10.3390/brainsci13050743/s1, Video S1: Control euploid mouse running at 18 cm per second; Video S2: Control euploid mouse running at 36 cm per second; Video S3: Ts65Dn mouse running at 18 cm per second; Video S4: Ts65Dn mouse running at 36 cm per second.

## Appendix A

**Table A1:** Factorial ANOVA statistics and post hoc comparison results for the measures depicted in Figure A2. Significant genotype dependence was found in all measures of variability. All *p*-values < 0.05 are written in bold.

Normalized Gait Dynamics Measure from Figure A2 (Panel)	ANOVA Results for Each Dependent Variable *F*_1,108_; *p* =			Post Hoc Pairwise Comparisons *p* = @18 cm/s; *p* = @36 cm/s			
	Speed	Genotype	Sex	Ts65Dn Female vs. Control Female	Ts65Dn Male vs. Control Male	Ts65Dn Female vs. Ts65Dn Male	Control Female vs. Control Male
Swing Percentage (a)	273.61; **<0.0001**	0.85; 0.3574	6.15; **0.0147**	N/A	N/A	N/A	N/A
Lateral Shift (b)	0.0745; 0.7853	0.0913; 0.7631	0.0000; 0.9947	N/A	N/A	N/A	N/A
Front/Hind Distance (c)	88.203; **<0.0001**	8.4596; **0.0044**	1.0436; 0.3093	0.3668; 0.0840	0.2550; **0.0412**	0.6618; 0.1936	0.6960; 0.1161
Hind/Front Offset (d)	0.028; 0.8674	9.589; **0.0025**	1.702; 0.1948	**0.0431**; 0.0519	0.4052; 0.2206	0.9063; 0.7952	0.1756; 0.2212
Front/Hind Skew (e)	0.8042; 0.37185	1.10640; 0.2952	0.0120;0.9130	N/A	N/A	N/A	N/A
